# Nonplantigrade Foot Posture: A Constraint on Dinosaur Body Size

**DOI:** 10.1371/journal.pone.0145716

**Published:** 2016-01-20

**Authors:** Tai Kubo, Mugino O. Kubo

**Affiliations:** 1 The University Museum, The University of Tokyo, Hongo, Bunkyo-ku, Tokyo, Japan; 2 Department of Natural Environmental Studies, Graduate School of Frontier Sciences, The University of Tokyo, Kashiwanoha, Kashiwa, Chiba, Japan; Raymond M. Alf Museum of Paleontology, UNITED STATES

## Abstract

Dinosaurs had functionally digitigrade or sub-unguligrade foot postures. With their immediate ancestors, dinosaurs were the only terrestrial nonplantigrades during the Mesozoic. Extant terrestrial mammals have different optimal body sizes according to their foot posture (plantigrade, digitigrade, and unguligrade), yet the relationship of nonplantigrade foot posture with dinosaur body size has never been investigated, even though the body size of dinosaurs has been studied intensively. According to a large dataset presented in this study, the body sizes of all nonplantigrades (including nonvolant dinosaurs, nonvolant terrestrial birds, extant mammals, and extinct Nearctic mammals) are above 500 g, except for macroscelid mammals (i.e., elephant shrew), a few alvarezsauroid dinosaurs, and nondinosaur ornithodirans (i.e., the immediate ancestors of dinosaurs). When nonplantigrade tetrapods evolved from plantigrade ancestors, lineages with nonplantigrade foot posture exhibited a steady increase in body size following Cope’s rule. In contrast, contemporaneous plantigrade lineages exhibited no trend in body size evolution and were largely constrained to small body sizes. This evolutionary pattern of body size specific to foot posture occurred repeatedly during both the Mesozoic and the Cenozoic eras. Although disturbed by the end-Cretaceous extinction, species of mid to large body size have predominantly been nonplantigrade animals from the Jurassic until the present; conversely, species with small body size have been exclusively composed of plantigrades in the nonvolant terrestrial tetrapod fauna.

## Introduction

Body size affects many aspects of biological phenomena in organisms; therefore, the evolution of body size is one of the central issues in evolutionary biology [1]. The evolution of body size has been investigated not only in extant animals, but also in extinct animals [2–7]. Because they were the largest terrestrial animals ever to live on Earth, the body sizes of nonavian dinosaurs (hereafter, simply referred to as dinosaurs, when including Mesozoic Avialae we use the term “volant dinosaurs”) have drawn considerable attention and have been reconstructed using various methods [8–12]. For example, the evolutionary pattern and distribution of body sizes among dinosaur species have been analyzed using large datasets [3,4,13,14]. Compared with those of other major terrestrial vertebrate groups, the body size distribution of dinosaur species exhibits three distinct features. First, the largest dinosaurs are heavier by an order of magnitude than any other terrestrial animals [15]. Second, when the number of species is plotted against log body mass, dinosaurs exhibit a distribution that is skewed toward a large body size; conversely, the distributions of other major vertebrate groups are typically skewed toward a small body size [14]. Third, the smallest species of dinosaurs was heavier than those of other major terrestrial vertebrate groups, such as mammals, birds and fossil mammals of the Mesozoic and the Cenozoic, by more than two orders of magnitude [14,16–18].

These three features have received varying degrees of attention from researchers. The extremely large body size of the largest dinosaurs has been one of the hottest topics in dinosaur research. The reason for their gigantism has been investigated in detail [15]. The second feature, the skew toward large body size in the distribution of dinosaur species' body size, received less attention [14,19,20]. Two hypotheses have been proposed for this unique skew: taphonomic and sampling biases on one hand [13,20], and competition between middle-sized dinosaurs and juveniles of large dinosaurs due to ontogenetic niche shifts on the other hand [19]. In contrast, the reason underlying the larger size of the smallest dinosaurs compared with other terrestrial vertebrate groups has only rarely been investigated. The processes of dinosaur miniaturization toward and within the bird lineage have been well studied, and it has been indicated that the lower body size limit of nonavian dinosaurs is about 1 kg [3,4]. The requirement for digesting cellulose has been proposed as a factor that may have maintained herbivorous dinosaurs at modest to large body sizes [21]. However, this cannot explain why there were no small omnivorous or insectivorous dinosaurs smaller than 1 kg.

Dinosaurs are terrestrial, with functionally digitigrade or sub-unguligrade posture [22,23] and Dinosauromorpha (including birds) and *Scleromochlus*, the closest relative of Dinosauromorpha, are the only animals that exhibited nonplantigrade foot posture during the Mesozoic era [2,23]. In contrast, Cenozoic terrestrial mammals exhibited various foot postures, including plantigrade, digitigrade, and unguligrade. Biomechanical studies have noted that nonplantigrade foot posture is more efficient for large body size owing to its lower locomotor cost and faster speed [24,25]. In small body sizes, these merits of nonplantigrades are lost, and plantigrade foot posture appears to have advantages primarily in its retention of digit functionality and stability during locomotion [2]. Previous studies have investigated the constraints of foot posture on body size distributions of North American and African nonvolant terrestrial mammals and have considered how foot posture affected the body size evolution of North American Cenozoic nonvolant terrestrial mammals [2,5,26]. These studies have shown that mammalian groups with different foot postures have different body size distributions. The upper size limit of plantigrades and the lower size limit of nonplantigrades have been found to correspond to a body size of approximately 1 kg. The body size distributions of plantigrades appear skewed toward small body sizes, whereas those of digitigrades and unguligrades are normally distributed. Furthermore, the fossil records of North American mammals indicate that, after the emergence of nonplantigrade carnivores, most terrestrial plantigrades were constrained to small body sizes (<1 kg), with no directional change (decrease or increase) in body size; conversely, the body size of nonplantigrade animals steadily increased following Cope’s rule [2,27]. However, in studies of dinosaur body size evolution and distribution, the effect of foot posture has not yet been considered.

Here, we test the following hypotheses. 1) The observation that the lower body size limit of nonvolant terrestrial nonplantigrades at 1 kg is not specific to North American and African nonplantigrade mammals, but common for all nonvolant terrestrial nonplantigrade animals (including terrestrial birds, mammals, and dinosaurs), regardless of continents, taxa, and geological age. 2) Foot posture is a factor that could be correlated with the unique body size distributions of dinosaurs, which are skewed toward large body size. 3) After the emergence of a nonplantigrade lineage (Dinosauromorpha + *Scleromochlus*) in the Middle Triassic, evolutionary patterns of body sizes differ between the nonplantigrade lineage and coexisting plantigrade lineages (therapsids and nonornithodiran archosauromorphs) similar to that which occurred after the emergence of nonplantigrade mammals in Cenozoic North America, with an increase in the body size of nonplantigrades and constraints imposed on the body size of plantigrades [2].

## Materials and Methods

### Data collection

Body mass data for extant mammals and nonvolant terrestrial birds were taken from PanTHERIA and the CRC handbook of avian body masses, respectively [17,18]. For birds, the body mass of flightless terrestrial birds, i.e., Palaeognathae (Ostrich and relatives) excluding tinamous, flightless Gruiformes (flightless rails), and *Strigops habroptilus* (kakapo), were included in the analysis (S1 Table). Nonvolant birds on predator-free islands do not need cursoliality for escaping from predators [28]; thus they inhabit environments which are not comparable to other nonplantigrade tetrapods in the present study. They were therefore excluded from the analyses. Aquatic and amphibious nonvolant birds, i.e., Sphenisciformes, Anseriformes, Podicipediformes, and *Fulica gigantea* (giant coot), were not included because foot posture only constrains the body size of terrestrial animals [5,25,27]. Categorizing foot posture for each mammalian species is not straightforward because foot posture characteristics are continuous among mammals, and nonplantigrade foot posture convergently evolved in different lineages [29]. As a result, the same species are sometimes categorized differently among researchers [5,29,30]. Here, we define an organism’s foot posture to be nonplantigrade if their heels are not in contact with the ground when they stop walking. Some large nonplantigrades have elephantine feet that possess heel pads. Following Lovegroove and Haines [5], we consider these animals as nonplantigrade, because elephantine feet have the same advantage as nonplantigrades over plantigrade feet, because their foot bones are oriented vertically with respect to the direction of Ground Reaction Force [31]. We judged foot posture at family level because there was not sufficient information for each genus. Therefore, 24 mammalian families were considered nonplantigrade (S2 Table). Other mammalian families were treated as plantigrades. We did not categorize families comprising both plantigrade and nonplantigrade species (i.e., Caviidae, Eupleridae, Herpestidae, and Viverridae) or families comprising saltatorial organisms (i.e., Macropodidae, Potoroidae, and Lagomorpha) as nonplantigrade, to retain the same standards of foot posture categorization for dinosauromorphs, in which no convincing evidence suggests the existence of families with plantigrade or saltatorial members [23].

To investigate the constraints of nonplantigrade foot posture on extinct mammals and assess sampling and taphonomic biases affecting fossils through comparison with extant descendants, the body sizes of extinct Nearctic mammals belonging to the nonplantigrade families (according to the definition included above) were collected from the dataset of Lovegrove and Mowoe [32].

Body mass data of Mesozoic dinosaurs included data taken from Benson et al. [4] and additional data for eight dinosaur species (S4 Table). The body size estimates of Benson et al. [4] are based on Campione and Evans [10], which uses circumference of limb bones. These methods have been criticized for overestimation of weights in comparison with estimates using volumetric methods, especially when the target taxa lie outside of the size range of extant taxa and/or no extant analogues exist in terms of mode of locomotion or physiology [33–35]. We adopt their method because it was used to estimate body mass of a large number of dinosaur species and also because the simplicity of the method allows us to calculate the weight of additional taxa. When obtaining data from Benson et al. [4], we used data in which the mass of facultative quadrupeds had been calculated as bipeds and eliminated data based on juvenile specimens. Species of Mesozoic volant dinosaurs that are widely considered as volant, i.e., avialan species except for aquatic or nonvolant forms, *Microraptor* (2 species), *Rahonavis*, *Anchiornis*, and *Xiaotingia* were grouped as Mesozoic volant dinosaurs separately from other nonvolant dinosaurs, to test how flight ability affects the constraint of foot posture on body mass. When more than one body mass datum existed for the same species, the body mass data of Benson et al. [4], which have been used in the phylogenetic comparative method, were adopted. The body masses of eight newly added dinosaur species were calculated from circumferences of propodial bones using the formulas given by Benson et al. [4]. The circumferences of the humerus and femur of *Scolosaurus cutleri* were directly measured from the specimen at Fukui Prefectural Dinosaur Museum (FPDM-V0000031). The circumferences of propodial bones of *Latirhinus*, *Proa*, *Nankangia*, *Trinisaura*, *Anzu*, *Eousdryosaurus*, and *Dreadnoughtus* were taken from the literature or calculated from the anteroposterior and mediolateral widths of the midshaft, measured from figures in the literature [4]. When the midshaft of the femur was clearly not the thinnest part of the femur owing to the fourth trochanter, anteroposterior and mediolateral length were measured from above or beneath the trochanter, whichever was thinner. Although there were several nondinosaur dinosauromorphs with well-preserved femora and humeri, the figures in the literature were unfortunately not sufficient for the calculation of circumference, were lacking views from different directions, or (for quadrupedal taxa) figures of the humerus and femur were not from the same individual. Therefore, we could not estimate weights of non-dinosaur dinosauromorphs. However, some nondinosaur dinosauromorphs were very small in their body size, and it is important to include them in searching for the lower body mass limit of nonplantigrades. Therefore, we used the femur length of nondinosaur dinosauromorphs (S5 Table) to infer their weights by judging whether their femora were longer or shorter than those of dinosaurs which weighed 500 g and 1 kg, as 500g and 1kg are thresholds body weights between nonplantigrades and plantigrades (see the results section).

To investigate changes in body size in response to the emergence of the first nonplantigrades in the Middle Triassic, the femur length of both plantigrade and nonplantigrade species from the Middle Triassic to Middle Jurassic was collected. The nonplantigrade lineage comprised dinosauromorphs and *Scleromochlus*, whereas the two plantigrade groups examined here are nonornithodiran archosauromorphs and therapsids. A database of tetrapod femur length presented by Sookias et al. [6] was used, with additional data from 19 species. Because foot posture constrains only terrestrial tetrapods [2], volant pterosaurs, amphibious species belonging to Phytosauria and Doswelliidae, and the aquatic *Qianosuchus* were omitted from the database of Sookias et al. [6], and the femur lengths of aquatic thalattosuchians were not added to the database. Taxa with stratigraphic midpoints between the Middle Triassic and Middle Jurassic were selected. In addition, the femur lengths of 17 species of Archosauromorpha and two therapsid species were added to the database based on direct measurements or the literature (S5 Table). The absolute ages of each taxon were revised on the basis of the International Chronostratigraphic Chart 2013 [36].

### Data analyses

#### Analyses of body mass distributions

To investigate the lower limit of body mass for nonplantigrade terrestrial tetrapods, the lightest species were found within each of four groups: nonvolant dinosaurs, nonplantigrade mammals, extinct Nearctic nonplantigrade mammals, and nonvolant terrestrial birds. For extant species, because our data set covers most of the species with known body mass, it is likely that the lower body mass limit is close to the actual lower limit for each group. On the other hand, in the case of extinct taxa, there is a well-studied sampling bias in the fossil record, and this is especially profound for dinosaurs (see the discussion section). The analyses presented herein are based on most of the species with known body mass at the current time, but future discoveries may considerably change the picture.

**Fig 1:**
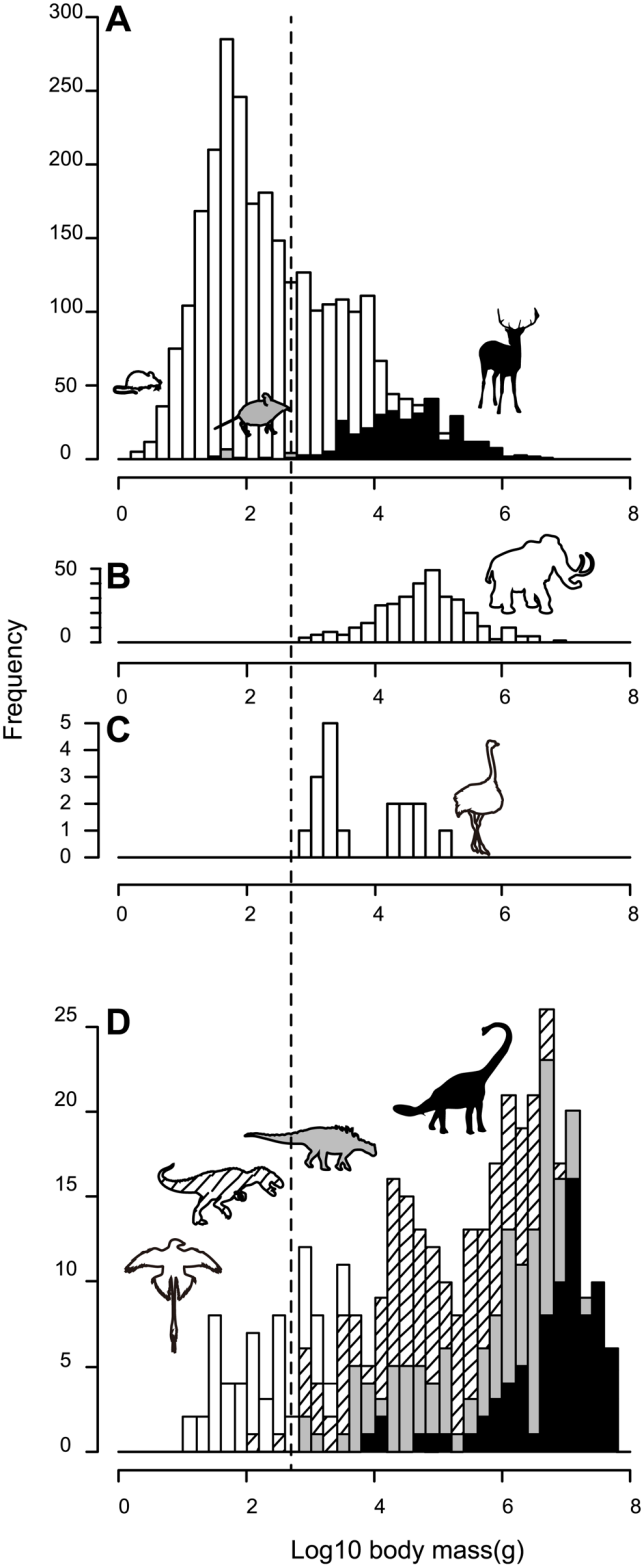
Body mass distribution for terrestrial tetrapod groups. Histograms for extant mammals of all foot postures (A), extinct Nearctic nonplantigrade mammals (B), nonvolant terrestrial birds (C), and Mesozoic dinosaurs including avialans (D). The dotted line indicates 500 g. In the histogram of extant mammals (A), plantigrade species are colored in white, species of Macroscelididae (elephant shrew) are in gray, and other nonplantigrade species are in black. In the histogram of dinosaurs (D), volant dinosaurs are in white, theropods are hatched, ornithischians are in gray, and sauropodomorphs are in black. Representatives of each group are shown as silhouettes on histograms. Except for Macroscelididae and two dinosaur species, all terrestrial nonplantigrade species were above 500 g. Except for nonvolant dinosaurs, the distributions of terrestrial nonplantigrade groups are not significantly different from a normal distribution.

**Fig 2:**
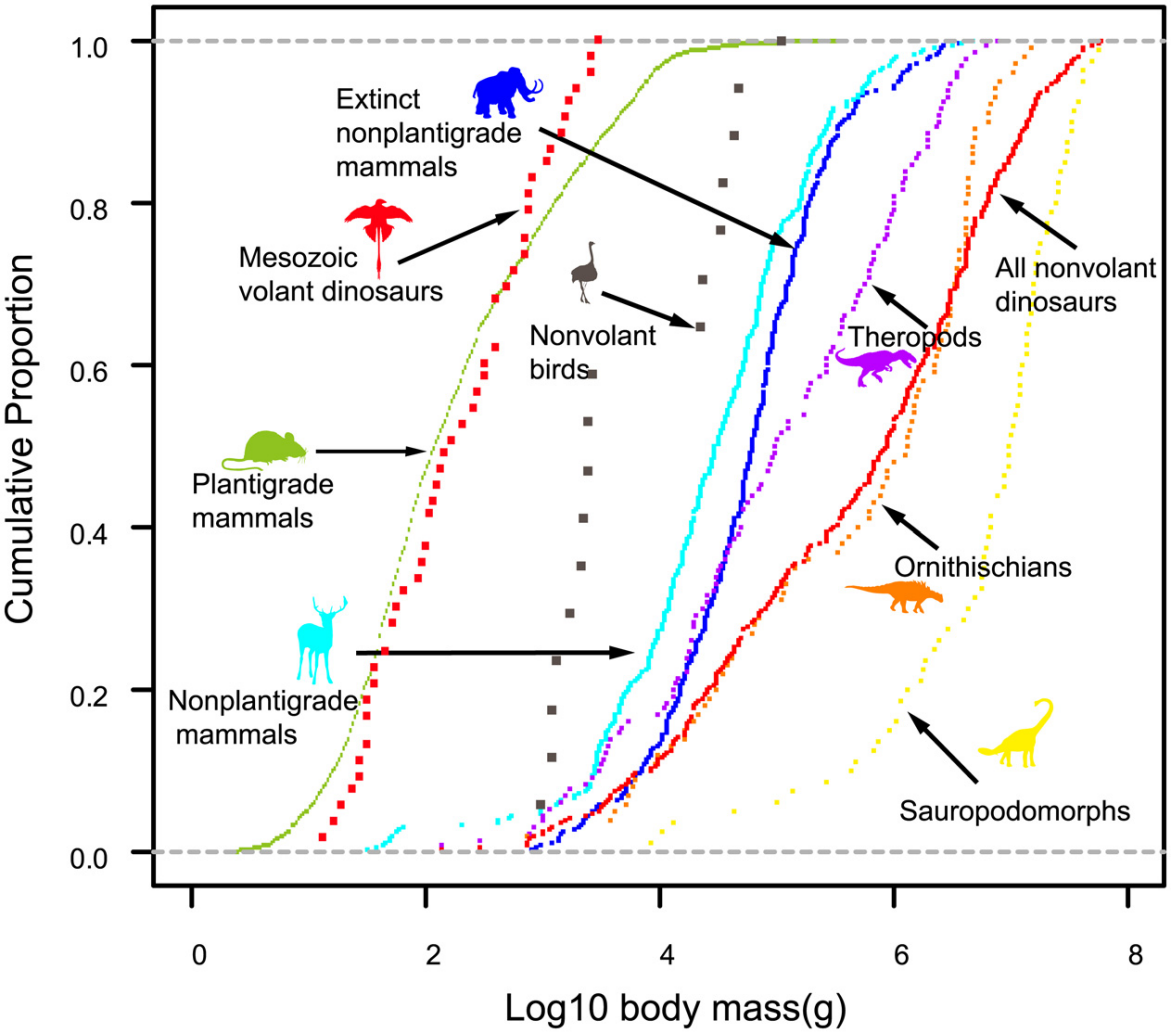
Cumulative curves of proportion of species number against log10 body mass for nonplantigrade taxa and plantigrade mammals. Each dot represents a species. Curves of plantigrade mammals and Mesozoic volant dinosaurs are shown on the far left and are distinct from curves for terrestrial nonplantigrade groups, indicating their considerably smaller body size. The curve for sauropodomorphs is on the far right, exhibiting a reverse L-shape that is distinct from other curves, indicating their large body size and lack of small species. Curves for extinct and extant nonplantigrade mammals are similar, although the curve for extinct species indicates a shift toward large body sizes. The difference between these two curves is likely due to taphonomic and sampling biases on the fossils of nonplantigrade mammals.

In analyzing body mass distributions, we followed the methods of O’Gorman and Hone [14]. The following 10 groups were analyzed: (1) all mammals regardless of foot postures (2,732 species) [17], (2) nonplantigrade mammals (330 species), (3) plantigrade mammals (2,402 species), (4) Nearctic extinct nonplantigrade mammals (326 species), (5) nonvolant terrestrial birds (17 species), (6) nonvolant dinosaurs (310 species), (7) Mesozoic volant dinosaurs (53 species), (8) nonvolant Theropoda (130 species), (9) Sauropodomorpha (80 species), and (10) Ornithischia (100 species) (Fig 1). Silverman’s test was conducted to count the number of modes and the location of each mode. A measure of skewness for each distribution was calculated. Kolmogorov–Smirnov tests were conducted to test if each distribution was significantly different from the normal distribution and from the body size distribution of other groups. In addition, to clarify the differences between these groups graphically, the cumulative frequency curves of plantigrade mammals, nonplantigrade mammals, extinct Nearctic nonplantigrade mammals, nonvolant terrestrial birds, nonvolant dinosaurs, Mesozoic volant dinosaurs, Theropoda, Sauropodomorpha, and Ornithischia were plotted (Fig 2).

#### Fitting evolutionary models on lineages with different foot postures

**Fig 3:**
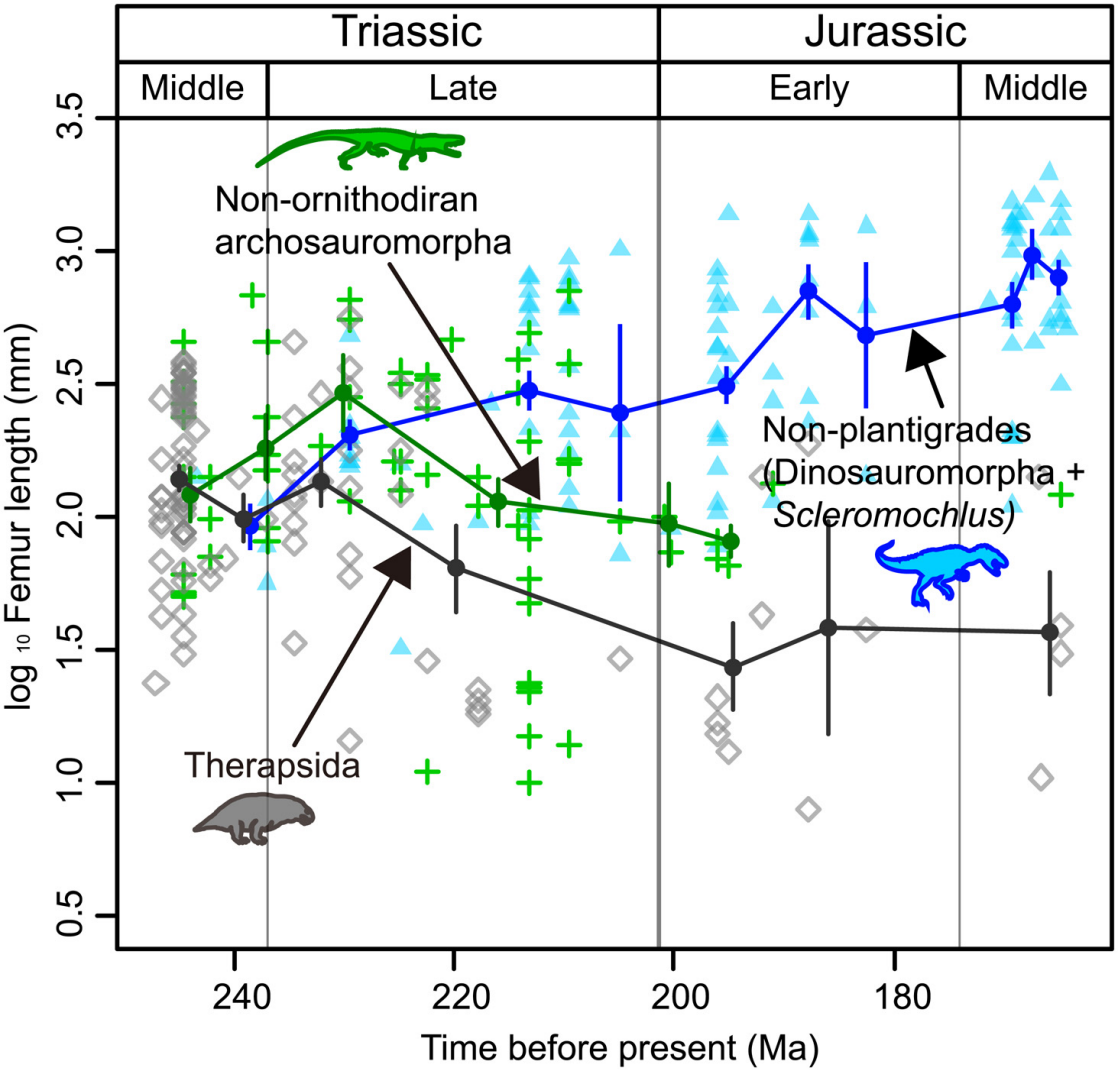
Femur length of terrestrial nonplantigrade and plantigrade lineages from the Middle Triassic to Middle Jurassic. Log femur length was plotted against the midpoint of the geological age estimated for each taxon. Mean and variance of femur length in each time bin are plotted. These values were used to fit evolutionary models (S9 Table). A trend toward longer femur length, reflecting a larger body size, was found in the nonplantigrade lineage (dinosauromorphs and *Scleromochlus*, blue triangle), whereas no directional trend was observed in plantigrade lineages (nonornithodiran archosauromorphs, green crosses; therapsids, gray diamonds).

Log femur lengths of Archosauromorpha and Therapsida were plotted against the midpoint of the age range for each taxon (Fig 3). Because these age ranges represent uncertainty in the dating of beds where fossils were found, rather than a real living range, the midpoints were selected for the evolutionary model fitting. The change in femur length patterns in three terrestrial lineages (i.e., nonplantigrade lineage of Dinosauromorpha + *Scleromochlus*, two plantigrade lineages of nonornithodiran Archosauromorpha, and Therapsida) were fitted to evolutionary models [37,38]. In addition, the lineage of Pseudosuchia was also analyzed, because plantigrade nonornithodiran Archosauromorpha was not a monophyletic group. The evolutionary model fitting used a sequence of mean and variance of femur length within each clade, without reference to phylogeny. Four evolutionary models were fitted to the sequence and their goodness of fit compared: a generalized random walk model (GRW), unbiased random walk (URW) model, an Ornstein–Uhlenbeck (OU) process, and stasis.

Body size was binned by stage(s) according to the midpoint geological age estimated for each taxon (S5 Table). There were several taxa for which the age range midpoint was 237 Ma, corresponding to the border between the Ladinian and Carnian. These taxa were binned into the Ladinian. When no data or only one datum were obtained for a given stage, this stage was analyzed with the dataset of the adjacent stage belonging to the same epoch because the analysis needed a variance that required at least two data sets for each time bin. Only one datum was found for after the early Jurassic (Sinemurian), for a nonornithodiran Archosauromorpha (*Sunosuchus* sp.) from the late Middle Jurassic (Callovian). Because of the long time gap, this data point was not included in the analysis; consequently, there were no age bins for nonornithodiran archosauromorphs and pseudosuchians younger than the Sinemurian. For therapsids, there were only four data in the Middle Jurassic; these data were grouped in the same time bin for analysis. The age of each time bin for the evolutionary model fitting was calculated by averaging the age of taxa included in each time bin, rather than using the midpoint of each time bin, because the temporal distributions of taxa were not even in most time bins (Fig 3). The mean and variance of femur length for each time bin used in the evolutionary model fitting were overlaid on the scatter plot, along with the trajectories of mean values for each lineage (Fig 3). In the analysis, joint parameterization was used because it has stronger statistical power in detecting the GRW model when the sampling error is high [38], which was the case here. Because Bartlett’s test showed that variance of femur length is significantly different between different time bins in all four lineages, pooled variance was not used [37]. For the statistical analyses, R 3.0.2 [39] and the packages silverman test, e1071, and paleoTS were used.

## Results

The lightest body mass for nonplantigrade mammals, nonvolant birds, and nonvolant dinosaurs in our data were 32.5 g, 957 g, and 123 g, respectively. In the case of Nearctic nonplantigrade fossil mammals, the lightest body mass was 827 g. These values are heavier by more than one order of magnitude than those for the smallest mammal and bird, which were found to be 2.3 g and 1.9 g, respectively [17,18]. Furthermore, among extant nonplantigrade mammals, only Macroscelididae mammals were lighter than 500 g. Among nonvolant dinosaurs, only two alvarezsauroid species were below 500 g (S3 and S4 Tables).

Although we could not calculate body mass, femur length is known for several nondinosaur dinosauromorphs and *Scleromochlus* (S5 Table). When their femur length was compared with that of dinosaurs, the body mass data for which were included in our data set, the femur length of *Scleromochlus* (32 mm) was shorter than that of *Parvicursor* (52.6 mm) [4], which was estimated to weigh 130 g. The femur length of *Marasuchus* was 56.3 mm, similar to that of *Parvicursor*. Other nondinosaur dinosauromorphs, such as *Lagerpeton*, *Dromomeron*, and silesaurids, had femurs longer than dinosaurs that weighed more than 1 kg, such as *Pantydraco*. Based on these comparisons, we considered that only *Scleromochlus* and *Marasuchus* would have had body mass below 500 g among nondinosaur dinosauromorphs, and assumed that other nondinosaur dinosauromorphs had weights above 1 kg.

In summary, among 983 species of terrestrial nonplantigrades for which body mass data have been collected here, only 13 species of macroscelid mammals and two species of alvarezsauroid dinosaurs weighed less than 500 g. Although not included in these 983 species, two species of nonplantigrade ancestors of dinosaurs, *Scleromochlus* and *Marasuchus*, were also terrestrial nonplantigrades with body masses lighter than 500 g. These data indicate that terrestrial nonplantigrades are typically heavier than 500 g, regardless of the taxa and geological age.

The skewness of body mass distribution indicates whether the group considered is skewed toward heavier body mass (negative values) or lighter body mass (positive values). For extant nonplantigrade mammals, nonvolant dinosaurs, and extinct Nearctic nonplantigrade mammals, skewness was found to be negative (−0.63, −0.46, and −0.03, respectively). The skewness of nonvolant birds and Mesozoic volant dinosaurs were found to be positive (0.363 and 0.076, respectively). The skewness values for nonvolant Theropoda, Ornithischia, and Sauropodomorpha were found to be −0.34, −0.69, and −1.55, respectively (S7 Table). However, except for nonvolant dinosaurs and two dinosaur clades (Ornithischia and Sauropodomorpha), all body mass distributions were not significantly different from the normal distribution (p > 0.05: S7 Table). Body mass distributions were significantly different between the groups analyzed here (p < 0.01, except for the comparison between nonvolant birds and nonplantigrade mammals, which p < 0.05), except for two comparisons that are between Mesozoic volant dinosaur and nonplantigrade mammals and between Mesozoic volant dinosaur and all extant mammals (S8 Table).

The evolutionary model fitting clarified that for the nonplantigrade lineage (dinosauromorphs + *Scleromochlus*), the best model was GRW with a positive step, indicating a trend toward larger body size in this lineage from the Middle Triassic to the Middle Jurassic. Other models were fitted poorly compared with GRW: the goodness of fit of other models were less than 1/8 of that of the best model. For plantigrade lineages, i.e., nonornithodiran archosauromorphs and therapsids, the best model was URW, which indicated that no trend in body size evolution existed among these two lineages during this time period. Nevertheless, other evolutionary models were not negligible for these two lineages, because the goodness of fit of the second best model was larger than 1/8 of that of the best model. The second best model was GRW with a negative step for therapsids, which indicated a steady body size decrease; conversely, for nonornithodiran archosauromorphs, the second best model is stasis that indicated an evolutionary optimum femur length of approximately 126 mm. The result of model fitting for pseudosuchians was almost the same as that of nonornithodiran archosauromorphs (S9 Table).

## Discussion

The body size data considered in the present study indicate that a lower size limit existed for terrestrial nonplantigrades, regardless of age and taxon. The lightest body mass for nonplantigrade mammals, extinct Nearctic nonplantigrade mammals, nonvolant terrestrial birds, and nonvolant dinosaurs were 32.5 g, 827 g, 957 g, and 123 g, respectively. These smallest nonplantigrades were an order of magnitude heavier than the smallest mammals and birds, which had masses of approximately 2 g [17,18]. The smallest nonplantigrades were species of the family Macroscelididae (elephant shrew), which is confined to Africa. Except for members of Macroscelididae, two species of alvarezsauroid dinosaurs (which were 130 g and 300 g) and likely two species of nondinosaur ornithodirans (*Scleromochlus* and *Marasuchus*) were below 500 g (See results section). Regardless of taxa or geological age, all other terrestrial nonplantigrades were above 500 g (Fig 1), which is larger than the median body mass of both mammals (182 g) and birds (41 g), based on a large body mass dataset [17,18]. The advantages of nonplantigrade foot posture in large body size, namely faster speed and lower locomotor cost, have been clarified on the basis of quantitative biomechanical comparisons with plantigrades, and the upper size limit of plantigrades (probably because of competition with or predation by nonplantigrades) has been highlighted often [5,24,25]. Our data indicate, at the same time, that plantigrade mammals occupied the small body size class (<1 kg) exclusively (Fig 1), and the body mass distributions of nonplantigrades indicate the existence of a pronounced body size barrier for nonplantigrades that lasted from the Mesozoic. Aves became free from this size barrier by evolving flight ability, and the body sizes of Mesozoic volant dinosaurs are also close to or lower than the 1 kg barrier (S4 Table, Figs 1 and 2). Nonplantigrade Macroscelididae species are known to have made networks of trails by removing bumps [40]. These examples suggest that the lower size limit of nonplantigrades is related to a lack of stability in nonplantigrade foot posture during ground locomotion [32], in which even small bumps can become significant obstacles for small animals (i.e., those with mass less than 1 kg).

Nonplantigrade foot posture cannot explain the skew toward large body sizes observed in nonvolant dinosaur species, because this skew is in contrast with the normally distributed body size characteristics of other nonplantigrades. Nevertheless, considering foot posture, rather than being skewed toward small body sizes (like other major vertebrate groups that contain species with various locomotor modes) as previously thought [14,19], the body size distributions of dinosaur species should exhibit a normal distribution (like other nonplantigrades).

Previous studies have attributed the skew toward large body size in dinosaur species body size distributions to taphonomic and sampling biases [20] or to oviparity and ontogenetic niche shifting of dinosaurs and the consequent occupation of small-sized niches by the juveniles of large dinosaurs [19]. The body size of theropods exhibits a normal distribution, when volant species and Avialae were excluded, as for other nonplantigrades, which is interesting considering that theropods have been sampled more intensely than other dinosaur taxa [20]. The body size of extinct Nearctic nonplantigrade mammals is generally larger than that of extant nonplantigrade mammals, but both are normally distributed, with similar cumulative curves (Fig 2 and S7 Table). Further, if Macroscelididae mammals that live only in Africa were excluded, body mass distributions of extant nonplantigrade and extinct nonplantigrade Nearctic mammals were only marginally different (p < 0.05 but > 0.01). If taphonomic and sampling biases for dinosaurs are similar to those for nonplantigrade mammals, they may not be sufficient to compensate for the differences between a normal distribution and the skewed distribution of dinosaurs, especially for sauropodomorphs, which have exhibited a body size distribution strongly skewed toward large body sizes (Figs 1 and 2 and S7 Table). Nevertheless, such taphonomic factors may affect dinosaurs more strongly than mammals owing to their older age and because, unlike mammals, dinosaurs cannot be reliably diagnosed and weighed based on an isolated tooth. The abundance of robust skull domes of small-bodied pachycephalosaurs compared with that of other similar-sized ornithischians exhibited preservational bias toward large and robust fossils and indicate that abundances of small-bodied dinosaurs (<100 kg) are strongly underestimated [41,42]. Also, considerable efforts have been made to find larger dinosaurs [43]. These two explanations, encompassing the taphonomic and sampling biases and unique ecology of dinosaurs, are not mutually exclusive; therefore, both factors may contribute to the negatively skewed body mass distribution of dinosaurs. Further study is needed to investigate the difference in taphonomic effects on dinosaurs and mammals.

Since nonplantigrade tetrapods first appeared in the Middle Triassic, their lineage (Dinosauromorpha + *Scleromochlus*) exhibited a steady and directional change toward larger body size, reflected in their femoral lengths, until the Middle Jurassic. During the same time interval, two main terrestrial plantigrade tetrapod lineages, therapsids and nonornithodiran archosauromorphs, exhibited no directional trend in body size (Fig 3 and S9 Table). These evolutionary patterns are equivalent to the body size evolution of mammalian lineages with different foot postures in North America during the Cenozoic after the emergence of nonplantigrade mammals: nonplantigrade lineages exhibited an increase in body size, whereas plantigrades were constrained to small body sizes with no directional body size change [2]. Directional evolutionary change is rarely found in fossil lineages [44]; however, in dinosaur lineages, directional body size increase is often supported [7, 27, 28] (see [6] for a differing opinion). It may be surprising that, in both the Mesozoic and Cenozoic, after the emergence of nonplantigrades, the body size of nonplantigrade lineages increased following Cope’s rule, while that of plantigrades did not [2]. However, this trend is convincing, because in our opinion the disparity in body size distribution between plantigrades and nonplantigrades would never have occurred unless trends in body size evolution differed between different foot postures.

Evolution toward a larger body size is often phrased as “success” or “prosperity,” especially when describing the early radiation of dinosaurs. However, regarding dinosauromorphs as an analogue of Cenozoic nonplantigrade mammals, radiation and extinction of dinosauromorphs can be understood more objectively. Body mass distributions indicate that nonplantigrades are typically restricted to large body sizes (>500 g), and small plantigrade species are much more abundant and diverse among modern mammals (Fig 1). In modern mammals, species with arboreal, semiaquatic, and fossorial locomotion are dominated by plantigrades, with nonplantigrades restricted to cursorial or graviportal locomotion. The nonplantigrade foot posture of dinosauromorphs may have prevented them from evolving body sizes smaller than 500g, the body size range that contains majority of extant mammalian species (Fig 1 and S3 Table), and left diverse vacant niches for other tetrapods, especially for mammals that exhibited ecological diversity similar to that of modern plantigrade mammals [47]. The nonplantigrade foot posture of dinosauromorphs may have allowed them to occupy mid to large body sizes in the fauna of the Jurassic and Cretaceous. Simultaneously, it would have prevented them from evolving small body sizes and the morphological diversity to match modern mammals [48]. The Cretaceous–Paleogene (K–Pg) extinction was size selective [16,49]. According to Fara [49], large-sized tetrapods (snout-vent length >150 cm) were significantly more likely to become extinct and medium-sized tetrapods (150 cm > SVL > 15 cm) showed higher extinction rate compared with small-sized tetrapods (15 cm > SVL) at the K-Pg extinction. The resulting lack of small-sized species because of the restrictions of the nonplantigrade foot posture made dinosaurs vulnerable to extinction. Avialae survived partly because of their small size (Figs 1 and 2), which they attained owing to their flight ability, which in turn allowed them to break the body size barrier of nonplantigrades. Although disturbed by the end-Cretaceous extinction event [2], among terrestrial tetrapod fauna, nonplantigrades have dominated the mid to large body size classes from the Jurassic until the present, while species with small body sizes have been exclusively plantigrades.

## Supporting Information

S1 File References for supplementary tables. (DOCX)

S1 Table Nonvolant terrestrial birds included in the analyses. (XLSX)

S2 Table Twenty-four Mammalian Families considered as nonplantigrade in this study. (XLSX)

S3 Table Log10 body size (g) of birds and mammals. (XLSX)

S4 Table Body size data of Mesozoic volant and nonvolant dinosaurs. (XLSX)

S5 Table Femur lengths of archosauromorphs and therapsids from the Middle Triassic to the Middle Jurassic. (XLSX)

S6 Table Dataset for evolutionary model fitting using the paleoTS module of R. (XLSX)

S7 Table Results of Skewness, Silverman's tests and tests for normality. (XLSX)

S8 Table Comparisons of body mass distribution between different groups by Kolmogorov-Smirnov Test. (XLSX)

S9 Table Results of the evolutionary model fitting using paleoTS module of R. (XLSX)
