# Supplementary material for: Nonplantigrade Foot Posture: A Constraint on Dinosaur Body Size
Source: PLoS One. 2016 Jan 20;11(1):e0145716. doi: 10.1371/journal.pone.0145716 (PMC4720450; doi:10.1371/journal.pone.0145716)
Supplement: S1 File — (DOCX) [file pone.0145716.s001.docx]

**References for supplementary tables**

50. Prieto-Márquez A, Serrano Brañas CI. *Latirhinus uitstlani*, a “broad-nosed” saurolophine hadrosaurid (Dinosauria, Ornithopoda) from the late Campanian (Cretaceous) of northern Mexico. Hist Biol. 2012;24: 607–619. doi:10.1080/08912963.2012.671311

51. Escaso F, Ortega F, Dantas P, Malafaia E, Silva B, Gasulla JM, et al. A New Dryosaurid Ornithopod (Dinosauria , Ornithischia) from the Late Jurassic of Portugal. J Vertebr Paleontol Palaeontol. 2014;34: 1102–1112.

52. McDonald AT, Espílez E, Mampel L, Kirkland JI, Alcalá L. An unusual new basal iguanodont (Dinosauria: Ornithopoda) from the Lower Cretaceous of Teruel, Spain. Zootaxa. 2012; 61–76. doi:10.11646/zootaxa.3609.5.8

53. Coria R a., Moly JJ, Reguero M, Santillana S, Marenssi S. A new ornithopod (Dinosauria; Ornithischia) from Antarctica. Cretac Res. Elsevier Ltd; 2013;41: 186–193. doi:10.1016/j.cretres.2012.12.004

54. Lacovara KJ, Lamanna MC, Ibiricu LM, Poole JC, Schroeter ER, Ullmann P V., et al. A gigantic, exceptionally complete titanosaurian sauropod dinosaur from Southern Patagonia, Argentina. Sci Rep. 2014;4: 1–9. doi:10.1038/srep06196

55. Lü J, Yi L, Zhong H, Wei X. A new oviraptorosaur (Dinosauria: Oviraptorosauria) from the late cretaceous of southern China and its paleoecological implications. PLoS One. 2013;8: e80557. doi:10.1371/journal.pone.0080557

56. Lamanna MC, Sues H-D, Schachner ER, Lyson TR. A new large-bodied oviraptorosaurian theropod dinosaur from the latest Cretaceous of western North America. PLoS One. 2014;9: e92022. doi:10.1371/journal.pone.0092022

57. Barrett PM, Butler RJ, Mundil R, Scheyer TM, Irmis RB, Sanchez-Villagra MR. A palaeoequatorial ornithischian and new constraints on early dinosaur diversification. Proc R Soc B Biol Sci. 2014; doi:10.1098/rspb.2014.1147.

58. Kammerer CF, Nesbitt SJ, Shubin NH. The first silesaurid dinosauriform from the Late Triassic of Morocco. Acta Palaeontol Pol. 2012;57: 277–284.

59. Maisch MW, Matzke AT, Stöhr H. *Sunosuchus* (Archosauria, Crocodyliformes) from the Toutunhe Formation (Middle Jurassic) of the Southern Junggar Basin (Xinjiang, NW-China). Geobios. 2003;36: 391–400. doi:10.1016/S0016-6995(03)00038-X

60. Trotteyn MJ, Martínez RN, Alcober OA. A new proterochampsid *Chanaresuchus ischigualastensis* (Diapsida, Archosauriformes) in the early Late Triassic Ischigualasto Formation, Argentina. J Vertebr Paleontol. Taylor & Francis; 2012;32: 485–489. doi:10.1080/02724634.2012.645975

61. Mukherjee D, Ray S. A new *Hyperodapedon* (Archosauromorpha, Rhynchosauria) from the Upper Triassic of India: implications for rhynchosaur phylogeny. Benson R, editor. Palaeontology. 2014; doi:10.1111/pala.12113

62. Sookias RB, Sennikov AG, Gower DJ, Butler RJ. The monophyly of Euparkeriidae (Reptilia: Archosauriformes) and the origins of Archosauria: a revision of *Dorosuchus neoetus* from the Mid-Triassic of Russia. Angielczyk K, editor. Palaeontology. 2014; doi:10.1111/pala.12110

63. Meng J, Hu Y, Wang Y, Wang X, Li C. A Mesozoic gliding mammal from northeastern China. Nature. 2006;444: 889–93. doi:10.1038/nature05234

64. Zhou C-F, Wu S, Martin T, Luo Z-X. A Jurassic mammaliaform and the earliest mammalian evolutionary adaptations. Nature. 2013;500: 163–167. doi:10.1038/nature12429
